# Prevalence of depression and anxiety in Colombia: What happened during Covid-19 pandemic?

**DOI:** 10.1371/journal.pone.0282760

**Published:** 2024-10-04

**Authors:** Sandra Martínez-Cabezas, Mónica Pinilla-Roncancio, Gabriel Carrasquilla, Germán Casas, Catalina González-Uribe

**Affiliations:** 1 School of Medicine, Universidad de Los Andes, Bogotá, Colombia; 2 Asiesalud, Bogotá, Colombia; 3 Fundación Santa Fe de Bogotá, Bogotá, Colombia; 4 Centre of Sustainable Development Goals for American Latina and the Caribbean (CODS); Polytechnic Institute of Viana do Castelo, PORTUGAL

## Abstract

The COVID-19 pandemic has impacted the well-being of millions of people around the globe. The evidence has shown that during the COVID-19 pandemic, the mental health of the population was affected, which means that there is an extra demand to implement different actions to mitigate and treat mental health disorders result of the pandemic. According to the literature it was expected that the prevalence of mental health disorders, such as anxiety and depression increased by 25 per cent worldwide, and Colombia was not the exception. However, there is not clear evidence on how much this increase might be. This study aims to estimate the prevalence of anxiety and depression for female and male adolescents and adults in Colombia before and during the COVID-19 pandemic. It estimated the potential increase of the prevalence in each group as a result of the COVID-19 pandemic in 2020. We used the Individual Registry of Health Services Delivery data from 2015–2021 to estimate the observed prevalence of anxiety and depression in Colombia for female and male adults. In addition, using the National Mental Health Survey 2015, we simulated the prevalence of anxiety and depression for adolescents (12 to 17 years) and adults (18 or older) in 2015 and using a static Monte Carlo simulation process we estimated the expected prevalence of depression and anxiety for each group from 2016 to 2021. The results of the analysis using revealed an important increase in the observed prevalence of depression and anxiety for adults and adolescents and men and women between 2015 and February 2020. When we simulated different scenarios using as a base line the National Mental Health Survey and estimated the prevalence of depression and anxiety for female and male adults and adolescents, we found that the prevalence of depression and anxiety has had an important increase in the last five years for all groups and had an important increase during 2020. This increase was greater for women compared to men, and adolescents than adults. Our results show the number of people who need potential attention from the health system in Colombia and highlight the importance to think about how to avoid and detect potential cases of anxiety and depression especially in female adolescents.

## Introduction

The COVID-19 pandemic has impacted the well-being of millions of people around the globe [1]. Throughout 2020–2021, different countries worldwide implemented strict quarantine measures to prevent the spread of the virus and avoid the saturation of health services. Lockdown measures, reduction of social interactions, restrictions to carry out physical activity, exposure to traumatic situations, such as loss of family members and friends, and constant exposure to information (e.g., recommendations, prohibitions, and misinformation) were elements associated with increased risk of developing mental health problems for individuals with and without a history of mental health problems [2–4]. The lockdown was an unprecedented moment given its magnitude on a global scale, vulnerable groups were affected, especially young people, due to the exposure to stressful conditions, isolation, and school lockdown [5, 6]. A survey conducted by the World Health Organization (WHO) in 130 countries showed that between June and August 2020, mental health services for vulnerable populations were affected. For example, disturbances were observed in 67% of psychological counselling services and psychotherapy; 30% of the countries reported difficulties in accessing medications for the treatment of mental disorders; and mental critical health services were disrupted in 93% of countries [7].

According to WHO, approximately one billion people globally live with a mental health disorder, yet the provision of mental health services is one of the most neglected and underinvested areas in LMICs [8]. Different studies have estimated the potential increase in the prevalence of anxiety and depression as a result of the pandemic; however, most of them have based their figures on non-representative data. Therefore, it is not possible to know the increase in the prevalence of these disorders because of the COVID-19 pandemic [9, 10]. Aiming to provide better estimators, Santomauro and collaborators [11] analyzed the change in the prevalence of major depressive disorders and anxiety disorders before and during the COVID-19 pandemic using data from surveys in a systematic literature review. According to the results of this study an increase of 53.2 million additional cases of major depression, and an additional 76.2 million cases of anxiety disorders is estimated worldwide. Using this information, the WHO estimated that anxiety and depression disorders might have increased by 25% during the COVID-19 pandemic, with women and children as the most affected groups [12].

The COVID-19 pandemic notably affected countries of the Americas, it was one of the regions with the highest number of cases and deaths in the world [13], and Colombia was one of the most affected countries in Latin America and the Caribbean. Indeed, more than five million cases and more than 120,000 deaths were reported by the end of December 2021 [14] and, the pandemic has generated negative impacts on the delivery of health services and the mental health of the population. The pandemic led to the formulation of strict quarantines and health services restrictions favouring elective consultation by telehealth, limiting health promotion and disease prevention activities [15, 16].

Colombia assessed the prevalence of mental disorders before the onset of the COVID-19 pandemic through the 2015 National Mental Health Survey. According to this survey, which provided insights into mental health conditions, 4.4% of adolescents aged 12–17 and 4.0% of adults aged 18 and older reported experiencing mental disorders in the year leading up to the survey. Mental health disorders were found to be more prevalent among residents of rural areas and individuals living in poverty [17].

To analyse the potential effect of the COVID-19 pandemic on the mental health of individuals in Colombia, some studies were conducted in 2020 and 2021 by different authors. One of the first analyses estimated the prevalence of mental health disorders using a web survey between April and May 2020 [18]. According to this study, 18% of the participants reported living with a mental health disorder, or that someone in their family was diagnosed with a mental health disorder in the previous six months to the survey; the results showed that women and individuals aged 18 to 29 were more affected by mental disorders in comparison with men and people from other age groups. Other researchers have also identified that women, young and poor people are more vulnerable to mental health disorders [19, 20]. In addition, PSY-COVID-19, a study conducted in 30 countries using Patient Health Questionnaire-2 and Generalized Anxiety Disorder-2 instruments, found that 35% of respondents have a higher risk of depression, and 29% have a higher risk of anxiety [21]; this percentage is lower compared to the one found by Colombia´s National Planning Department, where 52% of households reported a deterioration in mental health in adult members [22].

At the time of conducting this research, beyond descriptive studies relying on non-probabilistic samples, there were no studies that have analyzed this topic in Colombia. Therefore, this study aims to accomplish two primary objectives: 1) provide a national estimation of how much the prevalence of mental health disorders has changed from 2015 to 2021 according to national health registers (observed prevalence), and 2) estimate different scenarios on how the prevalence of depression and anxiety might have changed during the COVID-19 pandemic in 2020 (expected prevalence).

## Materials and methods

### Data sources

#### National mental health survey

We used data from the National Mental Health Survey 2015, which is a national representative survey as well as at the regional levels of the country. The survey excluded individuals aged 65 years or older with a positive dementia screen using the Mini-Mental State Examination, as well as individuals over 12 years of age with cognitive limitations, institutionalized individuals, and people who did not speak Spanish [17]. The final sample included for this analysis was 1,754 adolescents aged 12 to 17 years and 10,870 adults 18 years and older. We excluded children from 7 to 11 years old because the information was provided by the primary caregiver and it was not collected directly from the child.

#### Individual Registry of Health Services Delivery

The Individual Registry of Health Services Delivery (IRHSD) is the administrative register of healthcare providers in Colombia. It includes information on providers, consultations, and the provision of different services. Its main objective is to support the billing process of healthcare providers; therefore, the information is partial and does not include all providers or services delivered in the country. However, it is the only national register that includes information on the real use of healthcare services in the country. Thus, even though it is a limited data source, it is commonly used to analyse the demand for healthcare in the country [23].

The IRHSD includes the provision of services, classified according to ICD-10 diagnosis (International classification of diseases 10th). Information on the date of consultation, age, sex, region of residence, and type of service for everyone that has had access to the system is collected [24], therefore, the IRHSD allows the analysis of the number of outpatient consultations by ICD-10 diagnosis, according to the type of service received.

We used IRHSD data from 2015 to 2021 to estimate the observed prevalence of anxiety and depression, with the assumption that everyone who consulted was included in the register. We analyzed outpatient (consultations) and inpatient (hospitalization and emergency services) cases disaggregated by diagnosis, sex, and age. The download date of the IRHSD was June 14^th^, 2022, and we used the number of people who attended because it is equivalent to the number of cases, given that the system computes singular people.

### Statistical analysis

#### Observed prevalence

To estimate prevalence from 2015 to 2021, we used the total number of cases reported in the IRHSD with ICD-10 codes F41.1-F41.2-F41.3-F41.8-F41.9 for anxiety and F32.0- F321- F322- F323- F328- F329- F330- F331- F332- F333- F334- F338- F339- F341 for depression. As denominator, we used the population projections done by the National Administrative Department of Statistics (DANE), by sex and groups of age between 2015 and 2021 [25], then, we computed 95% confidence intervals. To estimate the percentage change in prevalences we used this formula: ((*V*_*2*_*-V*_*1*_*)/V*_*1*_) *100, where *V*_*1*_ represents initial value and *V*_*2*_ represents the final value.

#### Simulation (Expected prevalence)

Using the National Mental Health Survey 2015, we simulated the expected prevalence of anxiety and depression for adolescents (12 to 17 years) and adults (18 or older) from 2016 to 2020. We used two primary sources of information to inform the decision modelling. First, the estimated prevalence of anxiety and depression in Colombia reported by Santomauro [11], and second, the observed prevalence of depression and anxiety that we calculated using the IRHSD for each year between 2016 and 2020. We used an arithmetic static Monte Carlo simulation model to estimate the expected prevalence. Other researchers have used these simulations in the context of mental health service planning [26, 27].

We first calculated the prevalence of depression and anxiety for adults and adolescents using the National Mental Health Survey (2015). We used questions that aimed to identify individuals who had suffered any depression or anxiety disorder during the 12 months before the data was collected. Using this information as a baseline (before the COVID-19 pandemic), we calculated the rate of relative annual growth (the difference between year 2 minus year 1 divided by year 1) of the prevalence of anxiety and depression using the IRHSD for adolescents, adults, men, and women, and we computed the prevalence of depression and anxiety for the years 2016 to 2019, assuming that the observed relative annual growth in IRHSD as the real relative annual growth of the prevalence of depression and anxiety.

**Fig 1:**
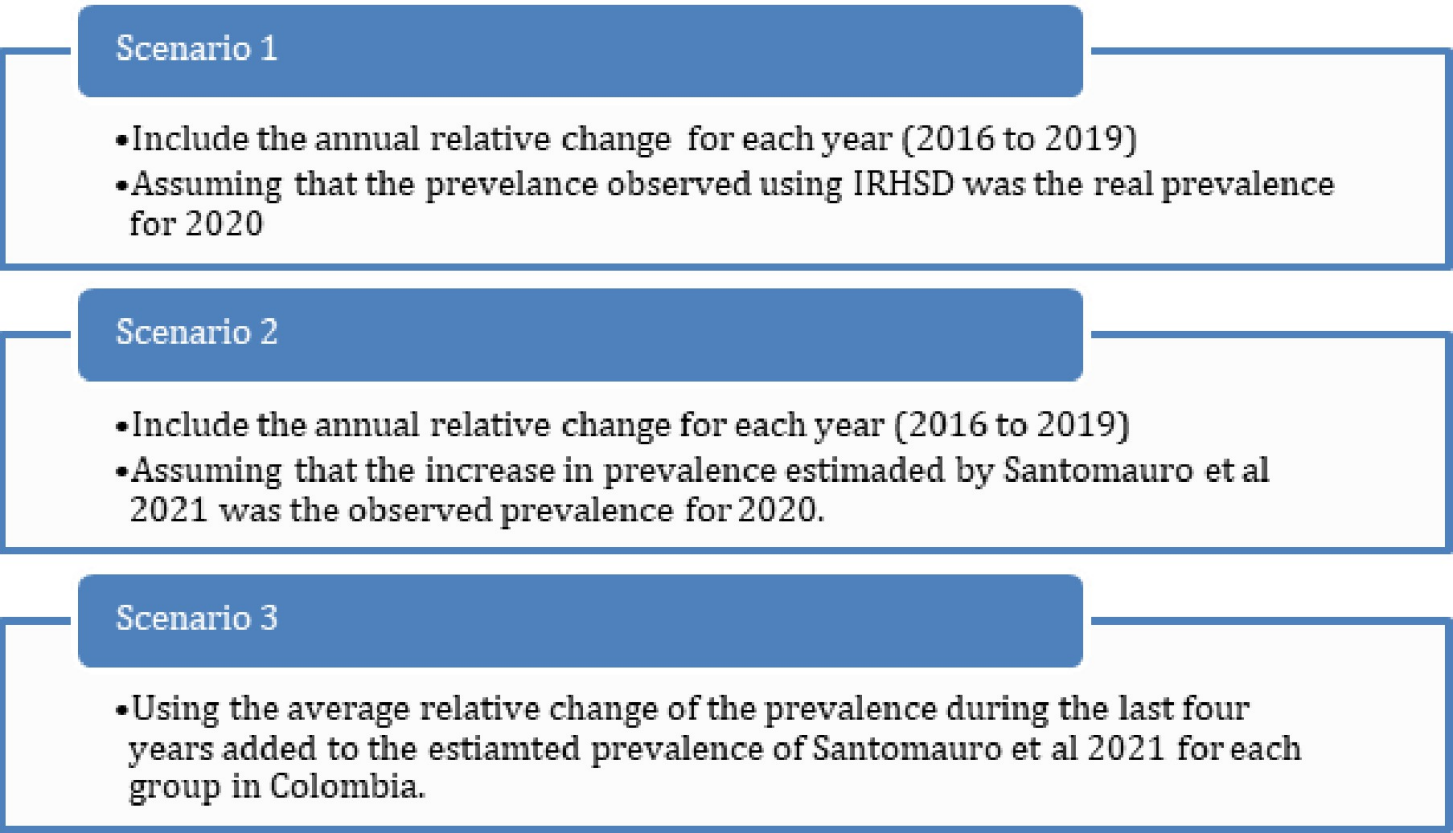
Scenarios used for the simulation of the prevalence of depression and anxiety.

To analyse the potential effect of the COVID-19 pandemic on the prevalence of depression and anxiety, we estimated three different scenarios for 2020 (Fig 1):

1. We assumed that the relative annual growth observed in IRHSD for 2020 was the real relative annual growth for 2020 (Scenario 1),
2. We used the average growth for depression and anxiety estimated by Santomauro [11]; during the COVID-19 pandemic for each group (men and women, adolescents, and adults) in Colombia (Scenario 2).
3. We assumed that the prevalence of depression and anxiety would have continued to change at a similar rate to the previous years. Therefore, we used the average percentage change in the prevalence between 2016 and 2019 to estimate the annual growth for 2020 and added to this percentage, the estimated increase in the prevalence presented by Santomauro [11] for Colombia (Scenario 3).

We computed each of the three scenarios for four groups: men 12 to 17, women 12 to 17 and men 18 years or older and women 18 years or older. For all the models we computed a simulation of 1000 iterations and calculated standard errors and confidence intervals. We used Stata 17® for all the analyses.

## Results

### IRHSD: Cases

**Fig 2:**
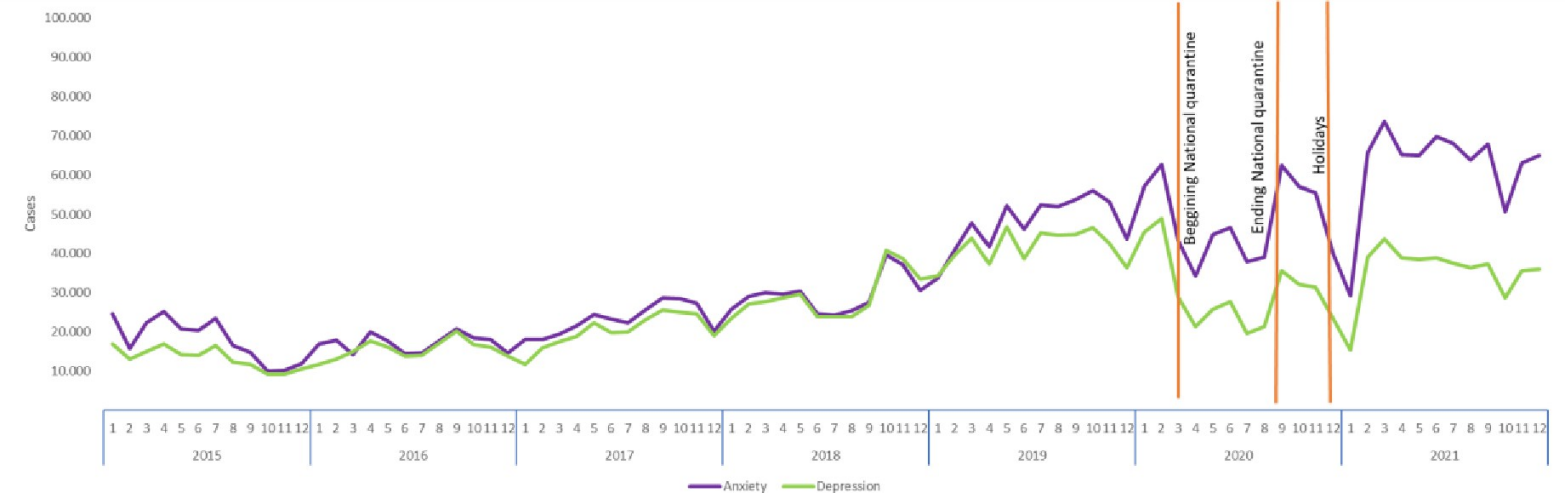
Number of cases per anxiety and depression disorders 2015–2021 by year and month in Colombia, all ages, all sexes. Source: Author’s own elaboration using data from the Individual Registry of Health Services Delivery 2016–2021.

Cases of both anxiety and depression in the IRHSD show similar trends between 2015 and 2018. In 2019, the number of anxiety cases increased, and this continued during January and February 2020 that accounted for more than 61,000 cases in each month, before the pandemic was declared. However, in March 2020 when the government established the national quarantine, the number of cases went down to 47,000 and increased again at the end of the first national lockdown in September 2020, to 66,000. In January 2021 the number of cases decreased to 31,000. Similar trends were observed in the case of depression, with a decrease from 48,000 cases in January 2020 to 31,000 cases in March 2020. In September 2020 (after the national lockdown), the number of cases increased to 37,000, and decreased again to 16,000 in January 2021, situation that might be associated with the implementation of local lockdowns (See Fig 2).

### Observed prevalence (using the Individual Registry of Health Services Delivery)

#### Anxiety

We observed an increase in prevalence of Anxiety since 2015, larger in female adolescents (0.12% in 2015 to 1.46% in 2021) and adult women (0.98% in 2015 to 2.61% in 2021). In all groups, except in adult males, during 2020, there was a reduction in the prevalence of anxiety. During 2021, the increase in the prevalence was higher than 50% in adolescents and more than 10% in adults, compared to 2020. See Tables 1 and 2 for details.

**Table 1:** Prevalence of anxiety and depression—Adolescents 12–17 years old.

	Women				Men			
Year	Anxiety	Annual % change	Depression	Annual % change	Anxiety	Annual % change	Depression	Annual % change
2015	0.12		0.05		0.14		0.07	
2016	0.12	2.24	0.08	57.34	0.14	-0.77	0.12	67.49
2017	0.20	71.98	0.16	109.29	0.20	44.40	0.14	21.30
2018	0.33	61.70	0.36	118.67	0.28	36.43	0.23	65.76
2019	0.70	112.79	0.84	135.13	0.49	79.24	0.46	94.64
2020	0.65	-6.86	0.67	-20.37	0.39	-21.26	0.31	-30.93
2021	1.46	123.84	1.37	106.49	0.62	60.50	0.49	56.06

Source: Author’s own elaboration using data from the Individual Registry of Health Services Delivery

**Table 2:** Prevalence of anxiety and depression—Adults 18 + years old.

	Women				Men			
Year	Anxiety	Annual % change	Depression	Annual % change	Anxiety	Annual % change	Depression	Annual % change
2015	0.98		0.75		0.42		0.29	
2016	0.91	-6.36	0.84	12.11	0.39	-7.92	0.32	8.90
2017	1.16	27.16	1.06	25.94	0.52	33.21	0.41	30.21
2018	1.40	20.25	1.42	34.11	0.68	31.99	0.62	49.17
2019	2.18	55.90	1.95	36.78	1.04	52.28	0.83	34.12
2020	2.14	-1.65	1.35	-30.46	1.07	2.98	0.59	-28.09
2021	2.61	21.53	1.46	7.75	1.21	13.50	0.63	6.19

Source: Author’s own elaboration using data from the Individual Registry of Health Services Delivery

#### Depression

The prevalence of depression had an increase between 2015 and 2021. In both, men, and women and in depression and in anxiety there is an increase up to 2019, then decrease in 2020 and an important increase in 2021. Female adolescents had the largest increase from 0.05% to 1.37%. A similar pattern occurred for adult women with an increase in depression of 1%. However, in this group (adult women) the largest prevalence was in 2021, with almost 3% of women aged 18 or older reporting cases of depression. For men (adolescents and adults), the prevalence of depression increased, but not in the same proportion as for women. Adolescent men there was an increase of 0.42 percentage points between 2015 and 2021. In adults the increase in 2021 was not as evident as for adolescents (See Tables 1 and 2).

#### Emergency and hospitalizations cases

We explored the number of cases in emergencies and hospitalizations to analyse the trends in the use of these services. As expected, we found similar trends to the ones for outpatient cases during 2020 in emergency cases between the beginning of the COVID-19 pandemic in March and the ending of the national quarantine on August 31^st^, 2020. The number of hospitalization cases decreased in March 2020 and rose in October 2020 for both, anxiety, and depression.

### Estimated prevalence: Monte Carlo simulation

According to the Mental Health Survey, in 2015, the prevalence of anxiety was 2.3% and 1.6%, for adult women and men, respectively. In the case of female and male adolescents, the prevalence of anxiety was higher in women (4.9%) than men (2.1%) (Table 3). In addition, the prevalence of depression in adults was higher for women (2.2%) compared to men (1.0%). In the case of male adolescents, the prevalence of depression was 0.7% in 2015 (Table 4).

**Table 3:** Estimated prevalence of anxiety in each simulated scenario.

Group	Base Line 2015	Increase 2020 by Lancet	Percentage change according to IRHSD 2020	Estimated result for 2020	SD	95% Confidence Interval		Estimated Population	Population 2020
**Scenario 1 **									
Women (18 years or older)	2.3%	33.2%	-1.7%	4.9%	0.2%	4.917%	4.925%	924,253	18,781,581
Men (18 years or older)	1.6%	28.0%	3.0%	3.4%	0.2%	3.417%	3.425%	591,702	17,296,667
Female adolescents (12 to 17 years)	4.9%	76.0%	-6.9%	12.6%	1.0%	12.549%	12.588%	298,868	2,377,905
Male adolescents (12 to 17 years)	2.1%	59.0%	-21.3%	4.02%	0.5%	4.011%	4.031%	99,405	2,472,245
**Scenario 2**									
Women (18 years or older)	2.3%	33.2%	-1.7%	6.8%	0.3%	6.847%	6.858%	1,287,053	18,781,581
Men (18 years or older)	1.6%	28.0%	3.0%	5.3%	0.3%	5.260%	5.272%	910,822	17,296,667
Female adolescents (12 to 17 years)	4.9%	76.0%	-6.9%	23.8%	1.7%	23.767%	23.830%	565,908	2,377,905
Male adolescents (12 to 17 years)	2.1%	59.0%	-21.3%	6.9%	0.8%	6.882%	6.912%	170,506	2,472,245
**Scenario 3 **									
Women (18 years or older)	2.3%	33.2%	-1.7%	7.8%	0.3%	7.806%	7.818%	1,467,180	18,781,581
Men (18 years or older)	1.6%	28.0%	3.0%	6.2%	0.4%	6.176%	6.189%	1,069,342	17,296,667
Female adolescents (12 to 17 years)	4.9%	76.0%	-6.9%	32.3%	1.8%	32.317%	32.384%	769,274	2,377,905
Male adolescents (12 to 17 years)	2.1%	59.0%	-21.3%	9.0%	0.9%	8.953%	8.987%	221,752	2,472,245

Source: Author’s own elaboration using data from the National Mental Health Survey 2015

**Table 4:** Estimated prevalence of depression for women and men in the three simulated scenarios.

Group	Base Line 2015	Increased reported by Lancet 2021	Percentage change according to IRHSD 2020	Estimated result for 2020	SE	95% Confidence Interval		Estimated Population	Population Projections 2020
**Scenario 1 **									
Women (18 years or older)	2.23%	30%	-30%	4.0%	0.2%	4.04%	4.05%	759,998	18,781,581
Men (18 years or older)	1.03%	21%	-28%	2.9%	0.2%	2.88%	2.89%	499,688	17,296,667
Female adolescents (12 to 17 years)	1.70%	68%	-20%	15.4%	1.4%	15.36%	15.42%	365,967	2,377,905
Male adolescents (12 to 17 years)	0.70%	56%	-31%	2.7%	0.5%	2.70%	2.72%	66,883	2,472,245
**Scenario 2 **									
Women (18 years or older)	2.23%	30%	-30%	6.9%	0.3%	6.87%	6.88%	1,291,164	18,781,581
Men (18 years or older)	1.03%	21%	-28%	3.8%	0.3%	3.83%	3.84%	663,483	17,296,667
Female adolescents (12 to 17 years)	1.70%	68%	-20%	31.0%	1.9%	30.93%	31.00%	736,217	2,377,905
Male adolescents (12 to 17 years)	0.70%	56%	-31%	4.7%	0.7%	4.69%	4.72%	116,263	2,472,245
**Scenario 3 **									
Women (18 years or older)	2.23%	30%	-30%	8.8%	0.4%	8.79%	8.81%	1,652,784	18,781,581
Men (18 years or older)	1.03%	21%	-28%	5.7%	0.4%	5.67%	5.69%	982,615	17,296,667
Female adolescents (12 to 17 years)	1.70%	68%	-20%	46.2%	1.8%	46.20%	46.27%	1,099,351	2,377,905
Male adolescents (12 to 17 years)	0.70%	56%	-31%	5.8%	0.9%	5.78%	5.81%	143,177	2,472,245

Source: Author’s own elaboration using data from the National Mental Health Survey 2015

#### Anxiety

In the case of anxiety, it is observed that if we only consider the increase observed in IRHSD (scenario 1), the prevalence of anxiety in 2020 would have been 12.6% for female adolescents, 4.9% for adult women, 4.0% for male adolescents, and 3.4% for adult men. These prevalences assume that the decrease in the prevalence observed using IRHSD in all groups was the real prevalence for 2020. However, when we include the estimated potential impact of the COVID-19 pandemic on the increase of anxiety, we obtain that the prevalence for female adolescents could have reached 23.8%, which corresponds to more than 565,000 female adolescents with anxiety in Colombia (scenario 2). Although the increase for the other groups would not be as large as for female adolescents, in the case of adult women, the prevalence would have increased almost two percentage points compared to scenario 1, which corresponds to an additional 300,000 adult women. Finally, the results of scenario 3 show that the prevalence of anxiety for female adolescents could have been 32.3% in the case where the increase in the prevalence of anxiety had had followed the same growth as the one observed between 2016 and 2019. Under this scenario, male adolescents would have a prevalence of 9.0%, or an additional 221,752 male aged 12 to 17 years with anxiety.

In the case of adult women, the difference between the second and third scenario is less than one percentage point; however, in absolute numbers this represents more than 150,000 women with anxiety. Finally, male adults would have been the group with the lowest increase of prevalence of anxiety among scenarios. However, under scenario 3, the prevalence for this group might be equal to 6.2%, which correspond to almost 770,000 male adults in the country with anxiety in 2020 (Table 3).

#### Depression

In the analysed of the different scenarios on how the prevalence of depression changed since 2015, we found that when only using information from IRHSD, the prevalence of depression until 2019 presented an increase in all groups compared to 2015. However, in 2020 the prevalence of depression decreased in all groups, with female adolescents as the group with the highest reduction (-20%) compared to 2019. However, even when considering this reduction, the prevalence of depression for female adolescents had the greatest increase going from 1.7% in 2015 to 15.4% in 2020. which correspond to more than 365.000 female adolescents in the country (scenario 1).

When we analysed scenarios 2, which includes increases over time based on the observed data and the increase reported by Santomauro [11], we found that there was an increase in the number of individuals living with depression. Indeed, the increase in the prevalence of depression was greater for female adolescents (31%), followed by adult women (6.9%) and male adolescents (4.7%), percentage that contrast to the ones of scenario one which are lower than in scenario 1.

Finally. when we analysed the results of the simulation including the average relative change of the prevalence of depression between 2016 and 2019, assuming that the prevalence of depression would have been like to the one calculated by Santomauro [11] (the additional effect of the COVID-19 pandemic), we found that around 46.2% of female adolescents and might have depression, number that represents an increase of 30.8 percentage points compared to scenario 1 and more than 733,000 female adolescents (Table 4).

## Discussion

This article estimated the prevalence of anxiety and depression for female and male adolescents and adults in Colombia before and during the COVID-19 pandemic. The results revealed an important increase in the observed prevalence of depression and anxiety for adults and adolescents (men and women) between 2015 and February 2020. However, between March and August 2020, there was a decrease in the number of registered cases for both diagnoses, aspect that might be associated with the barriers that individuals faced when accessing to different mental health services as a result of the restrictions and lockdown imposed during the pandemic [16]. In addition, when we simulated three different scenarios using as a base line the information of the National Mental Health Survey 2015 and estimated the expected prevalence for both depression and anxiety in adults and adolescents, we found that the prevalence of depression and anxiety increased significantly in the last five years for all groups. This increase is expected to be greater for women than for men, and for adolescents than adults. Depending on the assumptions made the final relative and absolute change varies for each of the four groups (adult men, adult women, male and female adolescents), for example, in the case of female adolescents the prevalence of depression and anxiety could be as high as 46.2% and 32.4%, respectively.

One important aspect to highlight is the observed increase in the number of cases between 2016 and February 2020. This increase was observed in all groups, but adult women and female adolescents were the groups with the largest increase. According to the Institute for Health Metrics and Evaluation, in Colombia, depression and anxiety contributed 5.4 and 3.3% respectively, to the total of Years Lived with Disability, in 2019 [28], these are the two most common mental health diseases in the country and the ones with the largest contribution to the burden of disease. Therefore, it is expected that the number of people seeking mental health services will continue to rise over time, as observed before the pandemic.

The COVID-19 pandemic brought different social, economic, and health challenges. including the provision of healthcare services for non-COVID-19 related diseases, this is one of the possible explanations of why the observed prevalence of depression and anxiety reduced in 2020. Indeed, according to the WHO, 75% of the countries reduced the provision of healthcare services or even stopped completely [7] and so was in Colombia [29]. Additionally, there was an increase in the number and type of barriers that individuals faced to access healthcare services, starting with the mandatory quarantine that was established between March and August 2020. These barriers might be one of the main reasons why the number of cases diagnosed with depression and anxiety observed during 2020 decreased compared to 2019. However, this result does not mean that the real prevalence of depression and anxiety reduced during the pandemic, but instead that individuals did not have access to services as much as before.

According to our results, women and female adolescents are the groups with the highest number of cases. In 2020, in comparison with men and male adolescents, the differences in the number of cases for women were greater compared to previous years, reflecting that these groups have a higher need and seek for services more often. This is a similar finding to the one of the global burden of disease, which revealed that the burden of depression was 50% higher for females than for males [30], in countries such as China and Portugal, a pronounced impact on women’s mental health has been noted, particularly in terms of the prevalence of depression, anxiety, and post-traumatic stress [31, 32].

Anxiety disorders are the predominant mental health issue among adolescents and exhibit a higher prevalence among older adolescents compared to their younger counterparts. Research suggests that approximately 3.6% of individuals aged 10–14 and 4.6% of those aged 15–19 experience symptoms indicative of an anxiety disorder [33]. In addition, during the COVID-19 pandemic female adolescents were the group that presented the highest increase in mental health disorders [34]. The COVID-19 pandemic was a real challenge for adolescents, because of isolation, lack of daily routines, lack of access to health services and schools, and the lack of capabilities of resilience [35]. In addition, periods where children and adolescents were not able to attend schools were associated with a decrease in physical activity, more screen time and irregular sleep patterns [10], all these factors affecting the mental health of individuals in this group of age. The isolation may have an influence on psychiatric disorder onsets during adolescence. For some adolescents, the numerous deaths related to COVID-19- were their first experience with death, and home confinement was associated with an increase in intrafamily violence [36, 37].

Finally, the three scenarios estimated in this article aimed to explore how the prevalence of depression and anxiety might have increased since 2015 and as a result of the COVID-19 pandemic in Colombia. Unfortunately, only the National Mental Health Survey allows the estimation of the prevalence of mental health disorders such as depression and anxiety in the country. Therefore, there is not an updated estimator of the prevalence of this illness and more importantly of the characteristics of individuals who are living with depression and anxiety. Nevertheless, the results of the three scenarios suggest a potential increase in the prevalence of depression and anxiety of male and female adolescents, and male and female adults, these groups also presented an increase in the number of consultations in the last four years, which means that the prevalence of depression and anxiety has also increased over time. One group where the prevalence of mental health disorders was higher were women (adolescents and adults), this group is in an important risk of mental health diseases and polices in the country should prioritise plans and programmes to reduce the negative impact of these diseases in their general well-being.

One lesson of the increase observed and estimated of mental health problems is the need to implement measures considering all the potential implications of those in the lives of individuals. During the COVID-19 pandemic different isolation strategies were used by governments to reduce the number of cases. However, little or no discussion took place analysing the potential effects of those decisions in the life of persons in the society, especially their mental health, thus, in future occasions it is important to acknowledge that health not only includes the physical health but also it is related to mental and psychological health.

### Strength and limitations

The results of this study contribute to the analysis and understanding of the mental health situation of individuals aged 12 years or older in Colombia. However, it is important to read the results carefully given data limitations related to the IRHSD. As mentioned before, IRHSD aims to support the payment of health providers; therefore, its main objective is not to collect data on the provision of services. Also, some public providers do not report information to IRHSD given that their payment is made through other systems, therefore there is a large probability that the information is not complete, and it only includes a segment of the population. Another limitation of the study is the use of the National Mental Health Survey of 2015, that even thought is the official and only source of information that allow the estimation of the prevalence of depression, anxiety and other mental health disorders, given that it is seven years old, it might not provide an accurate picture of the current situation of the country.

## Conclusions

The results of this study suggest that there has been an increase in the number of cases of anxiety and depression since 2016. In addition, the number of cases might have increased drastically because of the COVID-19 pandemic. No matter the escenario analysed or the assumptions that were made, the results revealed that there has been an increase in the prevalence of depression and anxiety, with a larger number of consultations and an expected higher prevalence for women and female adolescents. Therefore, the Colombian health system must rethink how mental health services are provided and how these vulnerable groups are prioritised. In addition, mental health aspects should be fundamental points in discussion in the health agenda, this topic should be included in the decision-making process during emergencies and strategies implemented to prevent mental health illness and how to detect potential cases of anxiety and depression, especially in female adolescents.
